# Good old days?

**DOI:** 10.1186/1742-5581-2-3

**Published:** 2005-04-13

**Authors:** Charles J Greenberg

**Affiliations:** 1 Harvey Cushing/John Hay Whitney Medical Library, Yale University, 333 Cedar Street, PO Box 208014, New Haven, CT, 06850-8014, USA

## Abstract

Alternative models of subsidizing scholarly publishing and dissemination have germinated and gathered momentum in the fertile soil of dissatisfaction. Like the stubborn spring dandelion that needs but a small crack in the sidewalk to flower boldly, the first flowers of Open Access in library literature, including *Biomedical Digital Libraries*, have sensed their opportunity to change the existing paradigm of giving away our scholarship and intellectual property, only to buy it back for the privilege of knowing it can be read. Will biomedical digital library and informatics researchers understand their role in a new era of Open Access simply by desiring an immediate uninhibited global audience and recognizing the necessity of open access peer-reviewed literature to become self-sufficient?

## Background

*The role that the publisher, librarian, and faculty will play in resolving the crisis has not been determined. Because of the potential impact of reduced collections on the mission of the health sciences center library, each group has a stake in working to contain the rise in scholarly journal prices and to stabilize the volatile journal market *[1].

These words, written in 1990 before the invention of the graphical web browser or Adobe Acrobat™ [2], predicted today's journal subscription-dominated budgets, but perhaps did not anticipate the astonishing library costs of maintaining both print and electronic scholarship simultaneously. Is it any wonder that alternative models of subsidizing publishing and dissemination have germinated and gathered momentum in the fertile soil of dissatisfaction? Like the stubborn spring dandelion that needs but a small crack in the sidewalk to flower boldly, the first flowers of open access in library literature, including *Biomedical Digital Libraries*, have sensed their opportunity to change the paradigm: giving away our scholarship and intellectual property, only to buy it back for the privilege of knowing it can be read. Will you, the biomedical digital library and informatics researcher, understand their role in a new era of Open Access simply by desiring an immediate uninhibited global audience and recognizing the necessity of open access peer-reviewed literature to become self-sufficient?

## Quality

*Biomedical Digital Libraries*, published by BioMed Central [3], is universally and freely available online to everyone; its authors retain copyright, and it is archived in at least one internationally recognised free repository [4]. *Biomedical Digital Libraries *however, has taken this further, by making *all *its content Open Access. In order to provide tangible benefits – no page or digital content limits or colour charges, unlimited image and data storage, preparation and inclusion in redundant global digital repositories – from 01 April, 2005, authors of articles accepted for publication in *Biomedical Digital Libraries *will be asked to pay an article-processing charge (APC) of £330 (€480, US$615). Authors from BioMed Central member institutions will not incur any APC. Many notable funding agencies authorize and encourage use of their grant resources to pay an APC [5]. The Editor-in-Chief will consider a limited number APC waiver requests per year on a case-by-case basis. Priorities for waivers include authors in countries served by the Health InterNetwork (HINARI)[6] or individual circumstance.

## Creating a New Tradition

Librarians and other information professionals are no doubt more familiar than most with the traditional access model: readers pay to view articles, either through library or personal subscriptions or by paying a fee each time they download an article. Escalating journal subscription costs have resulted in libraries subscribing to fewer journals [7]. Although scholarly journals traditionally publish authors' work for free (unless there are page or colour charges!) in exchange for obtaining permanent and exclusive rights to publish articles, subscriptions limit who and how many can immediately read, use and cite the research. However we feel about the level of profits from scholarly publishing, no one can deny that there are unavoidable costs in the quality production and timely distribution of peer-reviewed biomedical library research.

## A Time to Change

*Biomedical Digital Libraries*' Open Access policy changes the way in which peer-reviewed library research is published: following blinded peer review, all accepted articles are accessible online without the barrier of subscription or cost, and the author(s) retain copyright for their work and grant anyone the right to reproduce and disseminate the article [8]. Shortly thereafter, a copy of the full text of each article is permanently archived in an online repository separate from the journal. *Biomedical Digital Libraries*' articles are archived in PubMed Central [4], the US National Library of Medicine's full-text repository of life science literature, and also in repositories at the University of Potsdam [9] in Germany, at INIST [10] in France and in e-Depot [11], the National Library of the Netherlands' digital archive.

APCs will support the future publication of biomedical library and informatics articles with conscious attention to peer review and immediate impact on professional practice. open access to all of *Biomedical Digital Libraries*' articles. Although some authors may consider an APC of £330 (€480, US$615) to be an economic hardship, it must be remembered that *Biomedical Digital Libraries *does not levy additional processing charges on top of this fee. With the article being digital from birth, any amount of colour figures, photographs, and digitized video recording can be included with submissions.

## Open or Free?

Many libraries under increasing budgetary constraints feel indebted to websites like *Free Medical Journals *[13] which offers immediate assessment of which journals are releasing free content after a subscription embargo. Although a growing number of journals now offer delayed free access to their articles online, this is different from Open Access (as defined by the Bethesda Statement [14]). Journals with subscription revenue often delay free access for 6–12 months, and even when the full text is available, readers are not allowed to reproduce and/or disseminate the work because of restrictions imposed by the copyright policy. Biomedical Digital Libraries is not alone in the move to Open Access funded by APCs: the Public Library of Science (PLOS) has set up new Open Access journals, and have elected to set APCs of US$1500 for each accepted article, with institutional membership discounts available [15]. The high profile of PLOS in mainstream public media will raise awareness of Open Access and encourage researchers in all disciplines to understand and accept Open Access, with APCs as an acceptable revenue stream.

## It's your [copy] right!

By providing an unimpeded forum for authors that wish to maintain their intellectual property, APCs will enable Biomedical Digital Librariesto serve the individual content creator in the global library and informatics communities. We believe electronic publishing formats will benefit library and informatics practice and aid information science research, and we hope you will submit your next article to our protocol for blind peer review, which has without a doubt improved the organization and presentation of every submission so far.

## Declare Your Interest

More than 40 volunteer editorial staff members of *Biomedical Digital Libraries *solicit their peers and colleagues for evidence-based, quality research. In our editorial model, both authors and editorial reviewers declare their competing interests, especially any related to the APC. If you would like advice on what to declare, and how to declare it, please feel free to ask us prior to submission. Many authors have some form of competing interest, conscious or not. A declaration of a competing interest reminds the reader that an awareness of potential influence can help to distinguish an appearance of a relationship from actual manipulative intent.

## Competing interests

The Editor declares his affiliation with one of 519 BioMed Central institutional members in 40 countries that provide a reasonable alternative to direct individual APC revenue.

## Abbreviations

APC article-processing charge

HINARI Health InterNetwork

INIST INstitut de l'Information Scientifique et Technique (*Institute for Scientific and Technical Information*)

PLOS Public Library of Science
